# Characteristics of Our Patient Population

**DOI:** 10.1093/geroni/igab046.2025

**Published:** 2021-12-17

**Authors:** Kyrsten Hill

**Affiliations:** The University of Alabama, Tuscaloosa, Alabama, United States

## Abstract

To date, 106 patients have completed behavioral health assessments across three sites: a rural primary care clinic (n = 32), urban federally qualified health center (n = 33), and state-certified residential rehabilitation facility (n = 41). Patients ranged from 18 to 65 years of age (M = 38.6, SD = 11.4). Approximately 51% were female and 75% were non-Hispanic White (followed by 22% African American). Over 60% had a high school degree or less and found it at least somewhat difficult to pay for basic needs. Most patients endorsed substantial (44%) or severe (39%) drug use, with 40% endorsing opioid use. There were no significant differences in substance use by age group. Moderate to severe symptoms of depression (43%) and anxiety (49%) were common. Approximately 70% endorsed adverse childhood experiences, and 44% reported clinically significant post-traumatic stress symptoms. Measures of cognitive functioning and objective health literacy are currently being collected.

